# Multiple highly resistant clones of MRSA circulating among patients with skin and soft tissue infection, Peshawar, Pakistan 2021–2022

**DOI:** 10.1017/S0950268825100575

**Published:** 2025-09-16

**Authors:** Aman Ullah, Dorte Frees, Mujahida Mansoor, Shah Faisal Jamal, Jan Tkadlec

**Affiliations:** 1 Institute of Health Sciences, Kohat, Khyber Medical University, Peshawar, Pakistan; 2 Department of Veterinary and Animal Sciences, University of Copenhagen, Copenhagen, Denmark; 3 Department of Medical Laboratory Technology, Kohat University of Science and Technology, Kohat, Pakistan; 4 Institute of Paramedical Sciences, Khyber Medical University, Peshawar, Pakistan; 5 Department of Medical Microbiology, Charles University, Second Faculty of Medicine and Motol University Hospital, Prague, Czech Republic

**Keywords:** MRSA, Pakistan, *pvl*, SCC*mec* typing, *spa* typing

## Abstract

We aimed to determine the prevalence of antimicrobial resistance, carriage of Panton-Valentine leucocidin (PVL), and the clonal structure of MRSA isolates collected from skin and soft tissue infections at a tertiary care hospital in Pakistan. Between August 2021 and May 2022, 154 non-repetitive MRSA isolates were consecutively collected and characterized by antimicrobial susceptibility testing, SCC*mec* typing, *spa* typing, and detection of PVL by PCR. MLST clonal complexes (CCs) were inferred from *spa* type using the Based Upon Repeat Pattern (BURP) algorithm. High levels of resistance were observed to ciprofloxacin (85.7%), erythromycin (76.0%), sulfamethoxazole (68.8%), gentamicin (68.8%), fusidic acid (57.8%), tetracycline (55.8%), and clindamycin (42.2%). Clonal analysis revealed 16 lineages, with the most frequent being CC8-MRSA-IV (27.3%), PVL-positive “Bengal Bay” CC1/ST772-MRSA-V (26.0%), and CC1-MRSA-IV (16.2%). PVL was detected in 45.5% of isolates across multiple lineages. Our findings highlight the coexistence of high antimicrobial resistance and frequent PVL carriage among MRSA in Pakistan. Given the association of PVL with severe infections and the limited treatment options for multidrug-resistant strains, these data underscore a significant public health concern and the need for systematic surveillance and prudent antibiotic use.

## Introduction

Methicillin-resistant *Staphylococcus aureus* (MRSA) represents a major public health threat. It is responsible for a broad spectrum of infections in both hospitalized patients and individuals in the community, encompassing conditions from mild, self-limiting skin infections to severe and potentially fatal diseases such as sepsis and pneumonia [1]. Treatment of MRSA infection is often complicated by resistance to multiple antimicrobial agents [2] or by the production of virulence factors, like Panton-Valentine leucocidin (PVL), which is frequently found in community-associated MRSA (CA-MRSA) strains [3]. PVL is a bi-component exotoxin targeting human neutrophils, implicated in severe skin and soft tissue infections (SSTIs) and, in rare cases, necrotizing pneumonia [4]. In Pakistan, MRSA is a common cause of both hospital-acquired and community-acquired SSTIs [5]. A high prevalence of PVL has previously been reported among MRSA isolates from SSTIs in Pakistan, with diverse MRSA clones carrying PVL identified, including epidemic CA-MRSA lineages CC8-MRSA-IV (“USA300”) and ST772-MRSA-V (“Bengal Bay Clone”) [6].

A detailed description of the molecular structure of MRSA is essential to understand its global transmission and evolutionary dynamics [7]. Several molecular typing methods for MRSA typing have been developed, including multilocus sequence typing (MLST), SCC*mec* typing, and staphylococcal protein A (*spa*) typing. Among these, *spa* typing is particularly valued for its high discriminatory capacity, internationally standardized nomenclature, and relative cost effectiveness [8].

As data on the clonal structure of MRSA circulating in Pakistan are scarce, the aim of this study was to investigate the prevalence of distinct *spa* types, SCC*mec* types, and the presence of PVL among clinical MRSA isolates obtained from SSTIs at the Lady Reading Hospital (LRH), Peshawar, Pakistan.

## Methods

### Collection of MRSA isolates

Non-repetitive MRSA isolates were obtained from pus and/or wound swabs originating from SSTIs in patients attending both inpatient and outpatient departments at Lady Reading Hospital (LRH), Peshawar, Pakistan, between August 2021 and May 2022, at the Department of Pathology. Following culture on blood agar, presumptive *S. aureus* colonies were identified using the catalase test, mannitol fermentation, the tube coagulase test, and a latex agglutination test (Staphaurex, Thermo Scientific). Methicillin resistance was initially screened by the cefoxitin disc diffusion test, and the minimum inhibitory concentration for cefoxitin was determined by the E-test. The presence of the methicillin resistance determinant *mecA* was confirmed using multiplex PCR [9].

### Antimicrobial susceptibility testing

Susceptibility to ten commonly used anti-staphylococcal agents was determined by the disc diffusion method in accordance with the Clinical and Laboratory Standards Institute (CLSI) guidelines [10]. Isolates with intermediate susceptibility were classified as resistant. Quantification of multiresistance was based on the categorization of antimicrobial classes as defined for multidrug resistance in *S. aureus* [11].

### SCCmec typing

SCC*mec* typing of MRSA isolates (types I–VI) was performed using Multiplex PCRs as described previously by Kondo et al. [9] (Supplementary Figures S1 and S2). The PCR reactions were carried out in the final volume of 50 μl, containing 2 μl template DNA, 10 μl primer mixture (10 μM), 25 μl Dream Taq Green PCR Master Mix (2x), and 13 μl nuclease-free water. Amplification involved an initial denaturation at 94°C for 2 min, followed by 30 cycles of denaturation at 94°C for 2 min, annealing at 57°C for 1 min, and extension at 72°C for 2 min, with a final elongation at 72°C for 2 min. PCR products were separated on 1% agarose gel.

### spa typing and PVL detection

Multiplex PCR was employed to detect the PVL and *mecA* and *spa* genes following the protocol of Stegger et al. [12], with a modification (Supplementary Figure S3). Briefly, the PCR reaction was carried out in the final volume of 25 μl, containing 2 μl template DNA, 10 μl primer mix (10 μM), 11.5 μl of Qiagen Multiplex PCR Master kit, and 2.5 μl Milli-Q water. Amplification was performed in the T100 thermocycler (Bio-Rad, USA). The PCR products were separated on 1% agarose gel (E-Gel 48, Invitrogen, Grand Island, CA, USA).

*spa* typing was performed as described previously [13]. *spa* types of the MRSA isolates were determined using Ridom StaphType software; subsequently, all *spa* types were clustered into *spa*-Clonal Complexes (*spa*_CCs) using Based Upon Repeat Pattern (BURP) algorithm. MLST clonal complexes (CCs) were inferred from the results of *spa* typing.

### Statistics

Statistical analyses were conducted to assess differences in both continuous and categorical variables. For continuous variables such as age, the distribution of values between groups was compared. Specifically, the Mann–Whitney *U* test was used to compare the distributions of age between two independent groups (e.g., patient age in PVL-positive vs. PVL-negative isolates). When comparing age across more than two groups (e.g., across MRSA clonal lineages), the non-parametric Kruskal–Wallis test was applied. Patients were stratified into four age groups: children (0–14 years), youth (15–24 years), adults (25–59 years), and the elderly (60+ years). Categorical variables, including the proportion of PVL-positive isolates, the distribution of patients across age groups, resistance phenotypes, and sex distribution, were compared using the chi-squared test. When the expected frequency in any cell was less than five, Fisher’s exact test was used instead. All tests were two-tailed, and a *p*-value <0.05 was considered statistically significant.

### Ethic statement

The study was conducted as a laboratory-based investigation, and ethical approval or informed consent was not required. Basic demographic data (sex and age) were anonymized, and the result of the study has no impact on the patient treatment.

## Results

### MRSA isolates

Between August 2021 and May 2022, 154 non-repetitive MRSA isolates from inpatients and outpatients presenting with SSTI were collected. A total of 91 (59.1%) isolates were from males; and the median age of the patients was 24 years (ranging from 2 months to 86 years). Among the study population, there were 41 isolates from children (0–14 years), 37 from youth (15–24 years), 57 from adults (25–59 years), and 19 from elderly patients (60+ years). (Tables 1 and 2).

**Table 1.** Statistical analysis of patient age, gender, and antimicrobial resistance according to PVL status and clonal background

		PVL			Clonal background				Total (*n* = 154)
		Positive (*n* = 70)	Negative (*n* = 84)	*p* value	CC8-MRSA-IV (*n* = 42)	CC1/ST772-MRSA-V (*n* = 40)	CC1-MRSA-IV (*n* = 25)	*p* value	
Age (years)	Median (IQR)	24 (15–49)	24 (11–47)	0.363	19 (7–41)	24 (14–50)	29 (18–49)	0.228	24 (13–48)
	Children (0–14) (%)	17 (41.5)	24 (58.5)	0.463	15 (36.6)	10 (24.4)	4 (9.8)	0.1562	41 (26.6)
	Youth (15–24) (%)	19 (51.4)	18 (48.6)		7 (18.9)	11 (29.7)	7 (18.9)		37 (24.0)
	Adults (25–59) (%)	23 (40.4)	34 (59.6)		18 (31.6)	11 (19.3)	10 (17.5)		57 (37.0)
	Elderly (60+) (%)	11 (57.9)	8 (42.1)		2 (10.5)	8 (42.1)	4 (21.1)		19 (12.3)
Gender (%)	Males	42 (60.0)	49 (58.3)	0.834	25 (59.5)	23 (57.5)	13 (52.0)	0.832	91 (59.1)
	Females	28 (40.0)	35 (41.7)		17 (40.5)	17 (42.5)	12 (48.0)		63 (40.9)
Resistance (%)	Ciprofloxacin	60 (85.7)	72 (85.7)	1.000	36 (85.7)	38 (95.0)	22 (88.0)	0.360	132 (85.7)
	Erythromycin	51 (72.9)	66 (78.6)	0.409	34 (81.0)	30 (75.0)	20 (80.0)	0.789	117 (76.0)
	Sulfamethoxazole	48 (68.6)	58 (69.0)	0.949	33 (78.6)	31 (77.5)	11 (44.0)	**0.005**	106 (68.8)
	Gentamicin	51 (72.9)	55 (65.5)	0.325	35 (83.3)	34 (85.0)	11 (44.0)	**<0.001**	106 (68.8)
	Fusidic acid	30 (42.9)	59 (70.2)	**0.001**	32 (76.2)	16 (40.0)	20 (80.0)	**<0.001**	89 (57.8)
	Clindamycin	22 (31.4)	43 (51.2)	**0.015**	24 (57.1)	13 (32.5)	11 (44.0)	0.080	65 (42.2)
	Tetracycline	25 (35.7)	61 (72.6)	**<0.001**	33 (78.6)	10 (25.0)	21 (84.0)	**<0.001**	86 (55.8)
	Doxycycline	8 (11.4)	25 (29.8)	**0.006**	16 (38.1)	4 (10.0)	4 (16.0)	**0.007**	33 (21.4)
	Quinupristin/Dalfopristin	2 (2.9)	6 (7.1)	0.293	1 (2.4)	1 (2.5)	3 (12.0)	0.241	8 (5.2)
	Multiresistance*-median (IQR)	5 (4–6)	7 (5–7)	**0.002**	7 (5–8)	6 (4–6)	6 (4.5–7)	**0.008**	6 (4–7)
PVL positive (%)		–	–	–	7 (16.7)	40 (100.0)	0 (0.0)	**<0.001**	70 (45.5)

Demographic and antimicrobial resistance characteristics of MRSA isolates stratified by PVL status and by the three most common clonal lineages. Data are presented as numbers (%) for categorical variables and as averages (medians) for continuous variables. *p* values were calculated using chi-squared or Fisher’s exact test for categorical variables and Mann–Whitney *U* test or Kruskal–Wallis test for continuous variables, as appropriate. Multiresistance* is defined as non-susceptibility to at least one agent in three or more antimicrobial categories; the numbers represent the median number of antimicrobial categories to which the isolates were resistant. Statistically significant values are highlighted in bold.

IQR, interquartile range; PVL, Panton–Valentine leucocidin; MRSA, methicillin-resistant *S. aureus.*

**Table 2.** Distribution of antimicrobial resistance among MRSA isolates across different age groups

	Children (0–14 y; *n* = 41)	Youth (15–24 y; *n* = 37)	Adults (25–59 y; *n* = 57)	Elderly (60+; *n* = 19)	*p* value
Ciprofloxacin	39 (95.1)	31 (83.8)	46 (80.7)	16 (84.2)	0.193
Erythromycin	35 (85.4)	25 (67.6)	46 (80.7)	11 (57.9)	0.061
Sulfamethoxazole	32 (78.0)	25 (67.6)	34 (59.6)	15 (78.9)	0.187
Gentamicin	28 (68.3)	26 (70.3)	38 (66.7)	14 (73.7)	0.945
Fusidic acid	24 (58.5)	19 (51.4)	36 (63.2)	10 (52.6)	0.678
Clindamycin	19 (46.3)	13 (35.1)	26 (45.6)	7 (36.8)	0.673
Tetracycline	30 (73.2)	13 (35.1)	32 (56.1)	11 (57.9)	**0.009**
Doxycycline	12 (29.3)	9 (24.3)	9 (15.8)	3 (15.8)	0.393
Quinupristin/Dalfopristin	3 (7.3)	3 (8.1)	2 (3.5)	0 (0.0)	0.579
Multiresistance*-median (IQR)	6 (5–7.5)	5 (4–7)	6 (4–7)	6 (5–7)	0.340

The association between age group and resistance to each antibiotic was assessed using a chi-squared test (or Fisher’s exact test when expected counts <5). Statistically significant values are highlighted in bold.

IQR, interquartile range.

### Antimicrobial resistance

**Figure 1.**
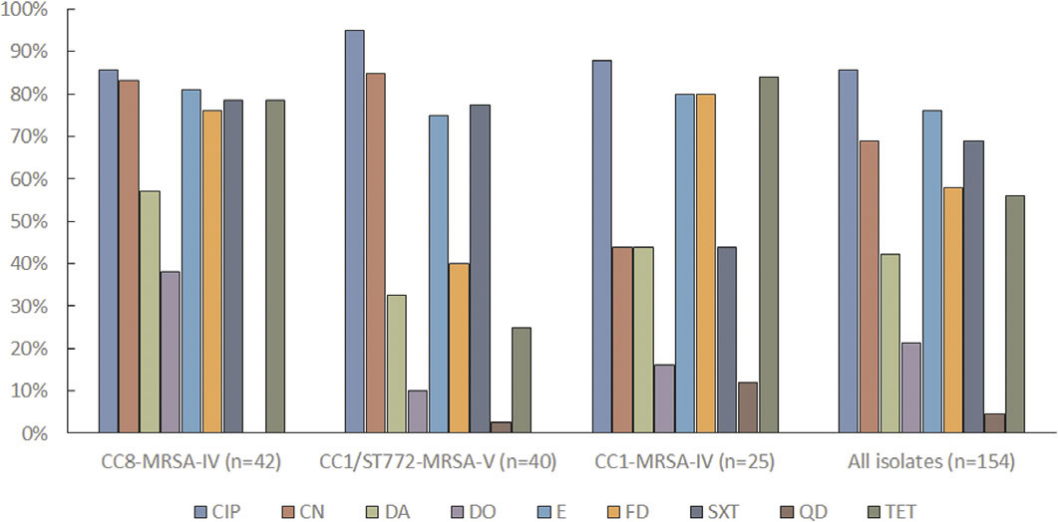
The proportion of resistant isolates among the major MRSA lineages, Peshawar, Pakistan 2021-2022. Frequencies of antimicrobial resistance in the three dominant clones in comparison with all isolates. Abbreviation: CIP, ciprofloxacin; CN, gentamicin; DA, clindamycin; DO, doxycycline; E, erythromycin; FD, fusidic acid; SXT, sulphamethoxazole; QD, quinupristin/dalfopristin; TET, tetracycline. No isolate was resistant to linezolid.

All isolates exhibited resistance to cefoxitin (MRSA), and the presence of *mecA* gene was confirmed by a multiplex PCR (Supplementary Figure S1). The MIC of cefoxitin ranged from 4 to >256 mg/L (Supplementary Table). Overall, a high level of resistance was observed. For six out of the ten antimicrobials tested, more than 50% of isolates were resistant. Isolates were frequently resistant to ciprofloxacin (*n* = 132; 85.7%), erythromycin (*n* = 117; 76.0%), sulfamethoxazole (*n* = 106; 68.8%), gentamicin (*n* = 106; 68.8%), fusidic acid (*n* = 89; 57.8%), and clindamycin (*n* = 65; 42.2%). Tetracycline resistance was present in 86 (55.8%) isolates; however, only 33 (21.4%) were resistant to doxycycline (Figure 1). Resistance to quinupristin/dalfopristin was rare (8 isolates; 5.1%), and no isolate was resistant to linezolid. The median number of antimicrobial classes to which the isolates were resistant was six. One isolate was resistant to all tested antimicrobials except linezolid. Conversely, three isolates were fully susceptible to all antimicrobials except for beta-lactams. Except for the high tetracycline resistance observed in children, there were no statistically significant differences in the distribution of resistance between age groups (Table 2).

### Clonal structure

**Figure 2.**
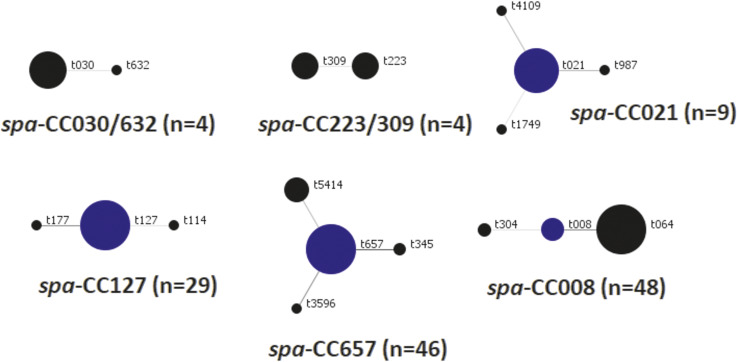
Cluster analysis of MRSA isolates based on similarity of their *spa* types. *spa*-Clonal Complexes (CC) were identified using (BURP) algorithm (Ridom StaphType software) that clusters *spa* types into groups, based on the similarity of their repeat patterns. The maximum cost for distances was four, and *spa* types shorter than five repeats were excluded. The founder of the *spa*-CC is indicated in blue. The size of the circle is proportional to the number of isolates within the corresponding *spa*-CC.

Among the MRSA isolates, four SCC*mec* types were identified: II (*n* = 4; 2.6%), III (*n* = 5; 3.3%), IV (*n* = 89; 57.8%), and V (*n* = 56; 36.4%). A total of 27 *spa* types, grouped into six *spa*_CCs (Figure 2 and Supplementary Table) and ten clonal complexes (CC1, CC1/ST772, CC5, CC8, CC8/ST239, CC22, CC30, CC88, CC121, CC361) were detected. Only three clonal complexes comprised more than 10 isolates: CC8 (*n* = 48); CC1/ST772 (*n* = 46); and CC1 (*n* = 32). These were assigned to 16 MRSA clones based on CC and SCC*mec* type. The three most frequent clones were CC8-MRSA-IV, CC1/ST772-MRSA-V, and CC1-MRSA-IV (Table 3). Statistical analysis indicated that CC1/ST772-MRSA-V isolates were resistant to fewer antimicrobial classes overall (*p* = 0.008). In particular, resistance to tetracycline (*p* < 0.001) and fusidic acid (*p* < 0.001) was less frequent in CC1/ST772-MRSA-V than in other lineages. Conversely, resistance to gentamicin (*p* < 0.001) and sulfamethoxazole (*p* = 0.005) was more common in CC1/ST772-MRSA-V, as well as in CC8-MRSA-IV, than in CC1-MRSA-IV. No significant differences were observed between the three clones with respect to patient gender, age, or age group. (Table 1).

**Table 3.** Clonal analysis of MRSA, Peshawar, Pakistan, 2021–2022

MRSA clone	No. (%)	PVL	*spa_*CC	*spa* types (no.)
CC8-MRSA-IV	42 (27.3)	7	*spa*_CC008	**t008**(7); t064(33); t304(2)
CC1/ST772-MRSA-V	40 (26.0)	40	*spa*_CC657	**t345**(1); **t657**(26); **t3596**(1); **t5414**(12)
CC1-MRSA-IV	25 (16.2)	0	*spa*_CC127	t114(1); t127(21); t177(1); t398(1); t1784(1)
CC1-MRSA-V	7 (4.6)	1	*spa*_CC127	t127(6); **t273**(1)
CC1/ST772-MRSA-IV	6 (3.9)	6	*spa*_CC657	**t345**(2); **t657**(4)
CC30-MRSA-V	5 (3.3)	5	*spa*_CC021	**t021**(4); **t4109**(1)
CC22-MRSA-IV	4 (2.6)	0	*spa*_CC223/309	t223(2); t309(2)
CC8/ST239-MRSA-III	4 (2.6)	0	*spa_*CC030/632 and CC021	t030(2); t632(1); t987(1)
CC8-MRSA-II	4 (2.6)	4	*spa*_CC008	**t008**(4)
CC30-MRSA-IV	3 (2.0)	3	*spa*_CC021	**t021**(2); **t1749**(1)
Other and undetermined^a^	14 (9.1)	4	Various	t1309(3); t6100(3); t304(2); **t363**(2); t030(1); **t314**(1); t525(1); **t2526**(1)
Total	154	70		

The table shows the distribution of MRSA clones in the study, along with their corresponding *spa* clonal complexes (*spa*_CC), *spa* types, and the proportion of PVL-positive isolates*. spa* types of isolates carrying PVL are in bold.

a Other and undetermined clonal complexes detected in this study include: CC8-MRSA-V (t304); CC121-MRSA-V(t314); CC8/ST239-MRSA-V (t030); CC88-MRSA-IV(t2526); CC361-MRSA-IV (t1309); CC5-MRSA-IV (t6100); undetermined (t363; t525).

### CC8-MRSA

Overall, the most common MRSA clonal lineage detected was CC8-MRSA-IV (*n* = 42; 27.3%). The isolates belong to *spa* types t064 (*n* = 33), t008 (*n* = 7), and t304 (*n* = 2). PVL-negative t064 isolates resemble an American USA500 (CC8-MRSA-IV-t064) HA-MRSA clone, also by exhibiting a high level of antimicrobial resistance.

All seven t008 isolates were PVL-positive, resembling characteristics of the epidemic USA300 CA-MRSA clone. CC8-MRSA included four PVL-positive t008 isolates belonging to SCC*mec* type II and two PVL-negative t304 isolates with SCC*mec* type V.

### CC1/ST772-MRSA

This CC was represented by PVL-positive isolates (*n* = 40) resembling ST772-MRSA-V, the Bengal Bay Clone, and a group of six PVL-positive CC1/ST772 isolates sharing the same *spa* types (t657 and t345) and *spa*_CC657 as CA-MRSA Bengal Bay Clone isolates, however, carrying SCC*mec* type IV.

### CC1-MRSA

Distinct from the previous lineage, CC1-MRSA was represented by *spa* types belonging to *spa*_CC127: t114 (*n* = 1); t127 (*n* = 27); t177 (*n* = 1); t273 (*n* = 1); t398 (*n* = 1); and t1784 (*n* = 1). SCC*mec* type IV was detected in 25 isolates, none of which carried PVL, resembling the multidrug-resistant but PVL-negative CA-MRSA clone originating in the South-Eastern Europe (CC1-MRSA-IV-t127). Another six PVL-negative t127 isolates carried SCC*mec* type V, and a single PVL-positive t273 isolate was identified.

### Other MRSA lineages

The remaining lineages were represented by seven or fewer isolates (≤4%). These included CC22-MRSA-IV (*n* = 4), similar to the EMRSA-15 (HA-MRSA) or Gaza Clone (CA-MRSA); CC8/ST239-MRSA-III (*n* = 4), the Brazil/Hungarian clone (HA-MRSA); PVL+ CC30-MRSA-V (*n* = 5), Western Australia (WA) MRSA-124 (CA-MRSA); and PVL+ CC30-MRSA-IV (*n* = 3), the Southwest Pacific clone (CA-MRSA) (Table 1).

### PVL-positive isolates

Almost half of the isolates (*n* = 70; 45.5%) harboured PVL (Supplementary Table). The most common PVL-positive lineage was the Bengal Bay Clone (CC1/ST772-MRSA-V; *n* = 40), along with the related CC1/ST772-MRSA-IV (*n* = 6) and the USA300 clone (CC8-MRSA-IV; *n* = 7). Minor lineages among PVL-positive isolates included CC30-MRSA-IV/V (*n* = 8; t021, t1749, and t4109), CC8-MRSA-II (*n* = 4; t008), and isolates of t363 (*n* = 2), t273 (CC1-MRSA-V), t314, and t2526 (Table 1). PVL-positive isolates were statistically less resistant than PVL-negative isolates, showing an average resistance to five versus six antimicrobial classes, respectively. Specifically, PVL-positive isolates exhibited significantly lower frequencies of resistance to clindamycin, fusidic acid, and tetracycline. No significant differences were observed between the groups with respect to patient gender, age, or age group (Table 1).

## Discussion

A high prevalence of methicillin resistance among *S. aureus* isolates from clinical samples was reported in studies from South Asia. In Pakistan, the MRSA prevalence ranges from 36.1% to 61.8% [14–16] and in India from 22.6% to 80.8% [17]. Previous reports on MRSA epidemiology in South Asia have documented the presence and the co-occurrence of multiple epidemic clones, including ST239-MRSA-III as the major healthcare-associated MRSA (HA-MRSA) clone and ST772-MRSA-V and ST22-MRSA-IV as major clones among CA-MRSA [17]. However, the number of studies on the molecular epidemiology of MRSA in Pakistan remains limited, often involving a small number of isolates. Here, we present data on the molecular epidemiology of a large, recent collection of 154 SSTI MRSA isolates collected in Peshawar, Pakistan, during 2021–2022.

In the present study, we applied *spa* typing and SCC*mec* typing to characterize the clonal structure of MRSA. We identified 27 distinct *spa* types, including 16 that, to our knowledge, are reported for the first time in Pakistan (t304, t1309, t6100, t223, t309, t363, t114, t1749, t177, t1784, t2526, t273, t3596, t398, t4109, and t525). Using SCC*mec* typing, the isolates were assigned to 16 MRSA clonal lineages.

The highest number of isolates belonged to CC8-MRSA-IV (*n* = 42). This lineage comprised a group of PVL-negative isolates, predominantly represented by *spa* type t064 (*n* = 33), which exhibited relatively higher levels of antimicrobial resistance compared with other MRSA isolates in this study, suggesting a possible healthcare-associated origin. Notably, the same *spa* type was reported as dominant among 26 isolates from Rawalpindi/Islamabad hospitals during 2006–2008 [18]. However, more recent studies indicate that PVL-negative CC8-MRSA-IV was rare in Islamabad and Peshawar between 2015 and 2018 [19, 20]. The same characteristics – t064; SCC*mec* type IV; and resistance to gentamicin, tetracycline, and sulfamethoxazole-trimethoprim – are common in the USA500 clone, which is considered healthcare-associated [21] but exhibits higher virulence potential than typical HA-MRSA clones [22]. Another seven PVL-positive CC8-MRSA-IV isolates, all characterized by *spa* type t008, showed lower levels of resistance, with one isolate susceptible to all antimicrobials except β-lactams. These isolates resemble the epidemic USA300 CA-MRSA clone, the predominant CA-MRSA lineage in the United States [3], which has also been reported at high frequency in Central Europe [23] and northern Taiwan [24].

The second-most common clone in this study, PVL-positive CC1/ST772-MRSA-V (*n* = 40), exhibited resistance to fewer antimicrobials compared with the other dominant clones identified. Nevertheless, these isolates were still frequently resistant to multiple agents, including ciprofloxacin, gentamicin, erythromycin, and sulfamethoxazole/trimethoprim – a resistance profile otherwise uncommon among CA-MRSA [3]. This clone represents a common South Asian CA-MRSA lineage, known as the Bengal Bay Clone [25]. With a relatively low fitness cost, it has successfully combined the high virulence typical of CA-MRSA with resistance to multiple antibiotics, a trait more characteristic of HA-MRSA. Multiple introductions of this clone into other countries have been documented, often linked to travel or migration from South Asia [25]. Unusually, six CC1/ST772-MRSA isolates carried SCC*mec* type IV. One such isolate, harbouring an SCCmec IV composite element, was identified among MRSA collected in 2017 in Rawalpindi, Pakistan [26]. Interestingly, a large phylogenetic study of the ST772 lineage reported an isolate from Bangladesh, within the basal clade of the Bengal Bay Clone, which retained remnants of SCC*mec* type IV [25].

PVL-negative CC1-MRSA-IV (*n* = 25) was the third-most common MRSA in this study, represented mainly by t127. This clone is present, albeit rare, in Pakistan [19, 20]. It is a multidrug-resistant CA-MRSA, originating in South-Eastern Europe, where it is endemic in countries such as Germany, Italy, Ireland, and Romania and has more recently spread to the Middle East [27].

Among the minor lineages detected in this study, ST239-MRSA-III (*n* = 4) represents the globally disseminated HA-MRSA clone previously reported in Pakistan [18] and remains the predominant MRSA clone in Indian hospitals [28]. However, a recent study in hospitals in Afghanistan found it to be rare [29]. Four PVL-negative CC22-MRSA-IV were detected, resembling EMRSA-15, a common European HA-MRSA clone [30]. PVL-negative CC22-MRSA-IV (t223) has also been reported as CA-MRSA in Russia [31] and was recently found to be a common MRSA in a large multicentric study in India [28]. Two additional CA-MRSA clones detected in this study included PVL-positive CC30-MRSA carrying SCC*mec* IV (*n* = 3; Southwest Pacific CA-MRSA clone) and SCC*mec* V (*n* = 5; WA MRSA-124), which were recently reported as major MRSA lineages in Kabul, Afghanistan [29].

The prevalence of PVL in this study was relatively high (45.5%), while an even higher prevalence of 70.8% PVL-positive MRSA was reported in Kabul, Afghanistan [29]. In both studies, the isolates originated exclusively or predominantly (65.3%) from SSTIs. A high prevalence of PVL among MRSA from community-associated infections, mostly skin and soft tissue, was similarly reported in a European multicentric study [32], highlighting the general association of PVL with SSTIs and the community setting. Interestingly, in Pakistan, as many as 71.0% of bloodstream MRSA isolates were PVL-positive in a recent study [20], an observation that is otherwise uncommon among MRSA from infections other than community SSTIs [30, 33].

The high level of antimicrobial resistance observed among MRSA in this study, including atypically multidrug-resistant CA-MRSA clones such as CC1/ST772-MRSA-V and CC1-MRSA-IV, may be linked to the recent increase in linezolid consumption in Pakistan [34]. The excessive and inappropriate use of antibiotics in hospitals and by the community in Pakistan promotes the dissemination of multiresistant MRSA clones [35]. Antimicrobial consumption, measured as Defined Daily Doses per 1,000 inhabitants per day, nearly doubled between 2019 and 2021 in the wake of the COVID-19 pandemic, with the highest levels recorded in the Peshawar region [34], where the present study was conducted. Frequently used antimicrobials – mostly administered orally – include beta-lactams, macrolides, sulphamethoxazole-trimethoprim, fluoroquinolones, and tetracyclines [34], corresponding to the agents to which resistance was commonly observed in our isolates.

The limitations of this study include the inability to analyse potential epidemiological links between MRSA cases or to determine the community versus healthcare origin of the isolates due to the lack of detailed clinical data. Additionally, as the isolates were collected from a single hospital, the findings may not fully represent the nationwide clonal distribution of MRSA.

In conclusion, our study provides a snapshot of the fragmented picture of MRSA epidemiology in Pakistan and South Asia. Taken together with other regional studies, Pakistan and neighbouring countries appear to be hotspots for the emergence of highly virulent, multidrug-resistant MRSA clones circulating in both community and healthcare settings. This is likely facilitated by insufficient epidemiological measures, high antimicrobial consumption, and dense human populations. Although multiple introductions of these clones to other countries have been documented, local spread has so far been limited. With the projected rise in global antimicrobial use [36], such clones could potentially disseminate widely, posing serious health risks due to limited treatment options. To mitigate this threat, systematic and detailed surveillance of MRSA epidemiology and more prudent antibiotic use in the region are urgently needed.

## Supporting information

10.1017/S0950268825100575.sm001 Ullah et al. supplementary material 1 Ullah et al. supplementary material

10.1017/S0950268825100575.sm002 Ullah et al. supplementary material 2 Ullah et al. supplementary material

## Data Availability

All relevant data are provided in the tables and figures within the main manuscript and the Supplementary Material.

## Supplementary material

The supplementary material for this article can be found at http://doi.org/10.1017/S0950268825100575.
