# Supplementary material for: Multiple highly resistant clones of MRSA circulating among patients with skin and soft tissue infection, Peshawar, Pakistan 2021–2022
Source: Epidemiol Infect. 2025 Sep 16;153:e113. doi: 10.1017/S0950268825100575 (PMC12529422; doi:10.1017/S0950268825100575)
Supplement: Ullah et al. supplementary material 1 — Ullah et al. supplementary material [file S0950268825100575sup001.docx]

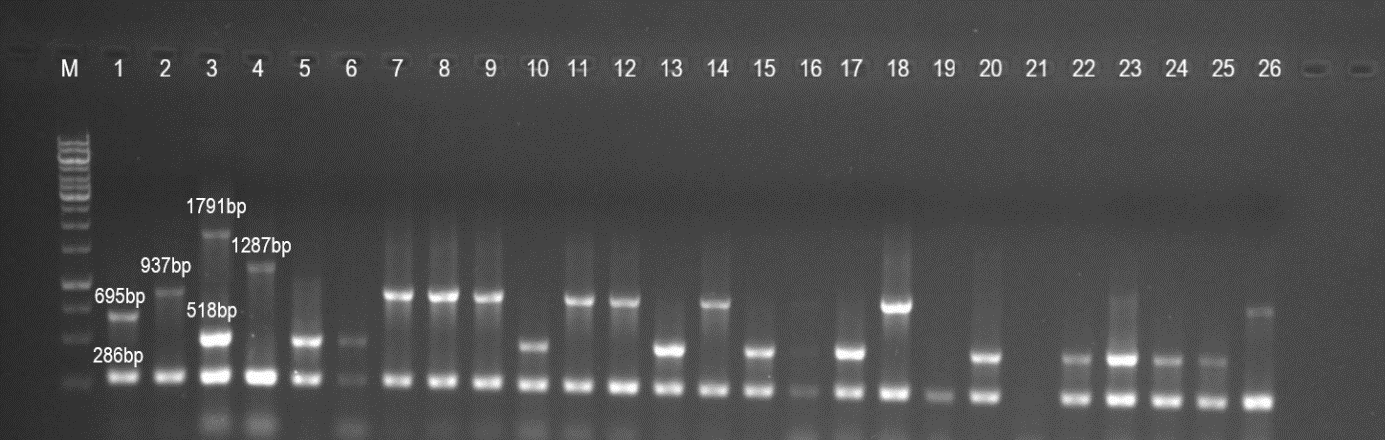


**Supplementary figure 1**. Multiplex PCR for SCC*mec* typing as by Kondo et al [9]. M-PCR_1 for identification of *ccr* gene complex (*ccrAB1*: 695 bp; *ccrAB2*: 937 bp; *ccrAB3*: 1791 bp; *ccrC1*: 518 bp; *ccrAB4*: 1287 bp); and detection of *mecA* (286 bp).

M: ladder 250-10000; 1-5: control strains (1: SCC*mec* type I; 2: SCC*mec* type II/IV; 3: SCC*mec* type III+SCCmercury; 4: SCC*mec* type VI; 5: SCC*mec* type V); 6 – 26: MRSA study isolates 1-4; 12; 15-20; 23-25; 77-82; 87. If no band was observed, the PCR was repeated for the corresponding isolate.


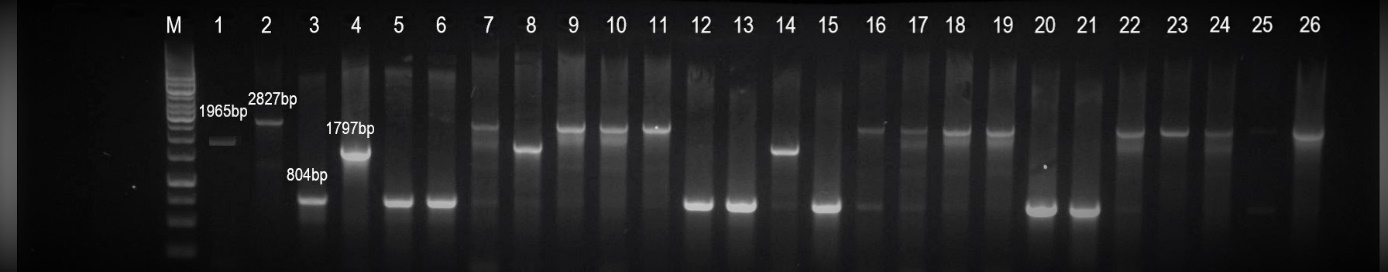


**Supplementary figure 2**. Multiplex PCR for SCC*mec* typing as by Kondo et al [9]. M-PCR_2 for the identification of *mec* gene complex class - class A (1965 or 1797 bp); class B (2827 bp); class C (804 bp).

M: ladder 100 - 3000 bp; 1 - 4: control strains (1: SCC*mec* type III; 2: SCC*mec* type IV; 3: SCC*mec* type V; 4: SCC*mec* type II); 5-26: MRSA study isolates 85-106. If no band was observed, the PCR was repeated for the corresponding isolate.


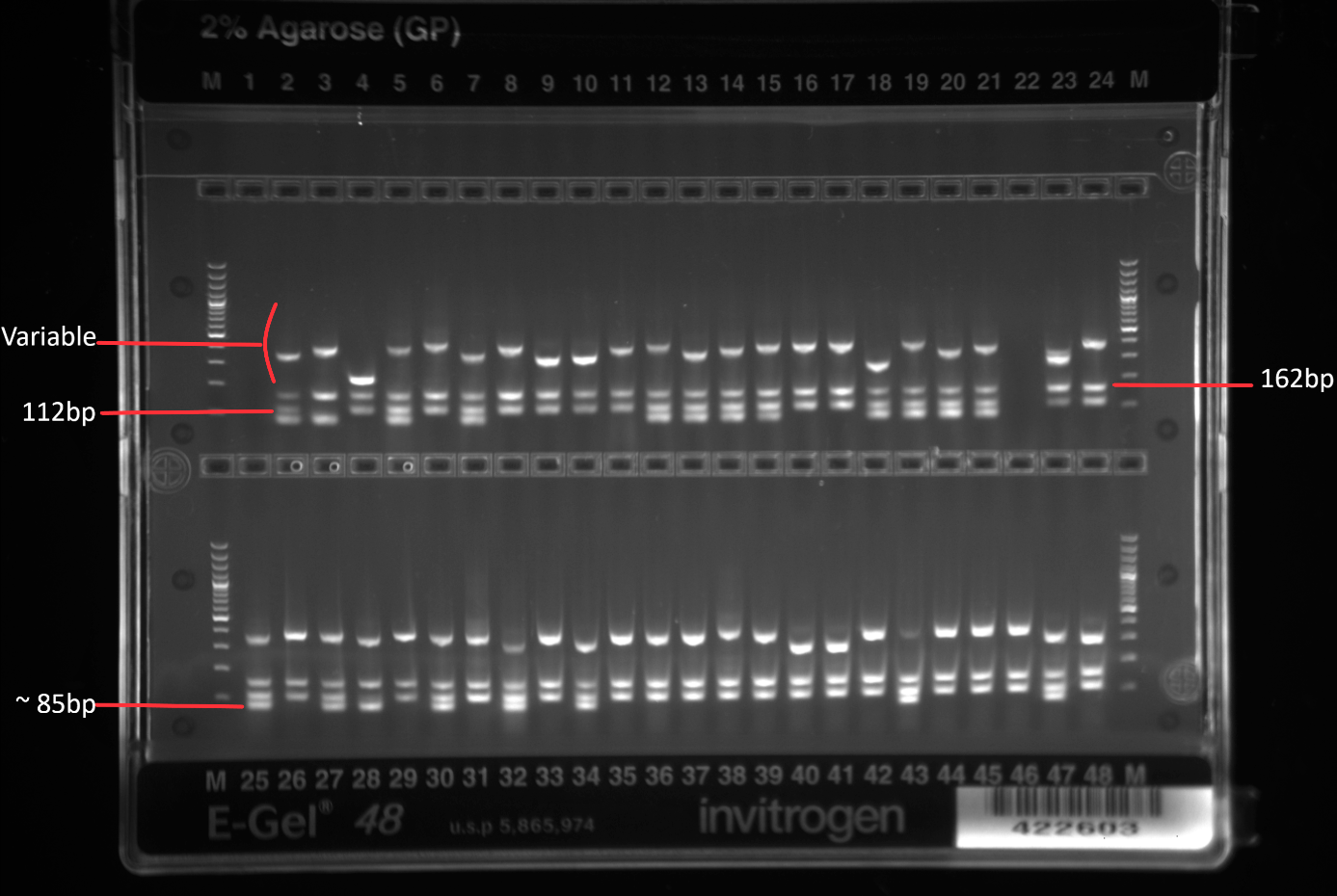


**Supplementary figure 3**. Multiplex PCR for detection of PVL (85 bp); *scn* (112 bp), *mecA* (162 bp), *spa* (variable: 200 -600 bp) adapted from Stegger et al 2012 [12]. Protocol was modified by adding scn detection using the primers scnF (ATATTTTGCTTCTGACATTTTCT) and scnR (AGCTACTGGAAGTTTAAACACT), results of *scn* detection are not reported in the study.

M: ladder 100 - 3000 bp; 1+22: negative controls; 2-21 and 23-48: MRSA study isolates 1-46.
