# Supplementary material for: Multiple highly resistant clones of MRSA circulating among patients with skin and soft tissue infection, Peshawar, Pakistan 2021–2022
Source: Epidemiol Infect. 2025 Sep 16;153:e113. doi: 10.1017/S0950268825100575 (PMC12529422; doi:10.1017/S0950268825100575)
Supplement: Ullah et al. supplementary material 2 — Ullah et al. supplementary material [file S0950268825100575sup002.docx]

**Supplementary table – MRSA isolates characterisation**

| **Isolate** | **Gender** | **Age (years)** | **Specimen** | **CIP** | **CN** | **DA** | **DO** | **E** | **FD** | **LZD** | **SXT** | **QD** | **TET** | **FOX MIC (mg/L)** | **Resistance profile** | ***Multiresistance** | **SCC*mec*  type** | ***spa* type** | ***spa*-CC** | **Clone** | **PVL** |
| --- | --- | --- | --- | --- | --- | --- | --- | --- | --- | --- | --- | --- | --- | --- | --- | --- | --- | --- | --- | --- | --- |
| 1 | M | 10 | wound swab | R | S | S | S | S | S | S | R | S | R | 12 | CIP-SXT-TET | 4 | V | t657 | *spa*-CC657 | CC1/ST772-MRSA-V | P |
| 2 | M | 53 | wound swab | S | S | R | S | R | R | S | S | S | S | 32 | DA-E-FD | 4 | IV | t008 | *spa*-CC008 | CC8-MRSA-IV | P |
| 3 | F | 50 | wound swab | R | S | S | R | R | R | S | S | S | R | 16 | CIP-DO-E-FD-TET | 5 | IV | t398 | excluded | CC1-MRSA-IV | N |
| 4 | F | 36 | wound swab | S | S | S | S | R | R | S | R | S | R | 16 | E-FD-SXT-TET | 5 | IV | t2526 | singleton | CC88-MRSA-IV | P |
| 5 | M | 23 | wound swab | S | S | S | S | S | S | S | R | S | S | 16 | SXT | 2 | IV | t223 | *spa*-CC223/309 | CC22-MRSA-IV | N |
| 6 | F | 4 | PUS | R | S | S | S | S | S | S | R | S | S | 8 | CIP-SXT | 3 | V | t657 | *spa*-CC657 | CC1/ST772-MRSA-V | P |
| 7 | M | 28 | wound swab | R | S | S | S | S | R | S | S | S | R | >256 | CIP-FD-TET | 4 | IV | t064 | *spa*-CC008 | CC8-MRSA-IV | N |
| 8 | M | 56 | wound swab | R | R | S | S | R | R | S | R | S | R | 16 | CIP-CN-E-FD-SXT-TET | 7 | IV | t127 | *spa*-CC127 | CC1-MRSA-IV | N |
| 9 | M | 45 | wound swab | S | S | S | S | S | R | S | R | S | R | >256 | FD-SXT-TET | 4 | IV | t127 | *spa*-CC127 | CC1-MRSA-IV | N |
| 10 | F | 30 | wound swab | R | R | R | S | R | R | S | R | S | S | 32 | CIP-CN-DA-E-FD-SXT | 7 | V | t5414 | *spa*-CC657 | CC1/ST772-MRSA-V | P |
| 11 | F | 47 | wound swab | S | S | S | S | S | S | S | S | S | S | 24 | Susceptible | 1 | II | t008 | *spa*-CC008 | CC8-MRSA-II | P |
| 12 | F | 18 | wound swab | R | R | S | S | R | R | S | R | S | S | 8 | CIP-DA-E-FD-SXT | 6 | V | t657 | *spa*-CC657 | CC1/ST772-MRSA-V | P |
| 13 | F | 80 | wound swab | S | S | S | S | S | S | S | R | S | R | 12 | SXT-TET | 3 | IV | t021 | *spa*-CC021 | CC30-MRSA-IV | P |
| 14 | M | 3 | wound swab | R | S | S | S | R | R | S | S | S | S | 24 | CIP-E-FD | 4 | IV | t008 | *spa*-CC008 | CC8-MRSA-IV | P |
| 15 | M | 50 | wound swab | R | R | S | S | R | R | S | R | S | R | 256 | CIP-CN-E-FD-SXT-TET | 7 | IV | t064 | *spa*-CC008 | CC8-MRSA-IV | N |
| 16 | M | 65 | PUS | R | R | R | R | R | S | S | R | S | R | 48 | CIP-CN-DA-DO-E-SXT-TET | 7 | IV | t064 | *spa*-CC008 | CC8-MRSA-IV | N |
| 17 | F | 18 | wound swab | S | S | S | S | S | S | S | R | S | S | 12 | SXT | 2 | V | t314 | singleton | CC121-MRSA-V | P |
| 18 | M | 35 | wound swab | R | S | S | S | S | S | S | S | S | S | 24 | CIP | 2 | II | t008 | *spa*-CC008 | CC8-MRSA-II | P |
| 19 | M | 60 | PUS | R | R | S | S | S | S | S | R | S | R | 32 | CIP-CN-SXT-TET | 5 | V | t657 | *spa*-CC657 | CC1/ST772-MRSA-V | P |
| 20 | M | 60 | wound swab | R | R | R | S | R | S | S | R | S | S | 32 | CIP-CN-DA-E-SXT | 6 | V | t5414 | *spa*-CC657 | CC1/ST772-MRSA-V | P |
| 21 | M | 35 | wound swab | R | R | R | S | R | S | S | S | S | R | >256 | CIP-CN-DA-E-TET | 6 | III | t030 | *spa*-CC030/632 | CC8/ST239-MRSA-III | N |
| 22 | M | 10 | wound swab | R | R | S | S | R | R | S | R | S | R | >256 | CIP-CN-E-FD-SXT-TET | 7 | IV | t064 | *spa*-CC008 | CC8-MRSA-IV | N |
| 23 | M | 12 | wound swab | R | S | S | S | S | S | S | R | S | R | 8 | CIP-SXT-TET | 4 | V | t021 | *spa*-CC021 | CC30-MRSA-V | P |
| 24 | F | 30 | wound swab | R | R | R | S | S | S | S | R | S | R | >256 | CIP-CN-DA-SXT-TET | 6 | IV | t064 | *spa*-CC008 | CC8-MRSA-IV | N |
| 25 | M | 18 | wound swab | S | S | S | S | S | S | S | S | S | S | 24 | Susceptible | 1 | IV | t008 | *spa*-CC008 | CC8-MRSA-IV | P |
| 26 | M | 60 | wound swab | R | R | S | S | S | R | S | R | S | S | 24 | CIP-CN-FD-SXT | 5 | V | t5414 | *spa*-CC657 | CC1/ST772-MRSA-V | P |
| 27 | M | 0,50 | wound swab | R | R | R | R | R | R | S | R | S | R | 24 | CIP-CN-DA-DO-E-FD-SXT-TET | 8 | IV | t064 | *spa*-CC008 | CC8-MRSA-IV | N |
| 28 | F | 55 | wound swab | R | R | S | S | R | S | S | S | S | R | 16 | CIP-CN-E-TET | 5 | IV | t008 | *spa*-CC008 | CC8-MRSA-IV | P |
| 29 | M | 18 | PUS | R | R | R | S | R | R | S | R | R | S | 16 | CIP-CN-DA-E-FD-SXT-QD | 8 | IV | t1309 | singleton | CC361-MRSA-IV | N |
| 30 | M | 14 | PUS | R | R | S | S | R | S | S | R | S | S | 8 | CIP-CN-E-SXT | 5 | V | t657 | *spa*-CC657 | CC1/ST772-MRSA-V | P |
| 31 | M | 0,25 | wound swab | R | R | R | S | R | R | S | R | S | R | >256 | CIP-CN-DA-E-FD-SXT-TET | 8 | IV | t064 | *spa*-CC008 | CC8-MRSA-IV | N |
| 32 | M | 18 | wound swab | R | R | S | S | S | S | S | R | S | S | 8 | CIP-CN-SXT | 4 | V | t657 | *spa*-CC657 | CC1/ST772-MRSA-V | P |
| 33 | F | 55 | wound swab | S | S | R | S | R | R | S | S | S | R | >256 | DA-E-FD-TET | 5 | IV | t064 | *spa*-CC008 | CC8-MRSA-IV | N |
| 34 | M | 28 | wound swab | R | R | S | R | R | R | S | R | S | R | >256 | CIP-CN-DO-E-FD-SXT-TET | 7 | IV | t064 | *spa*-CC008 | CC8-MRSA-IV | N |
| 35 | F | 41 | wound swab | R | R | R | S | R | R | S | R | S | S | 32 | CIP-CN-DA-E-FD-SXT | 7 | IV | t064 | *spa*-CC008 | CC8-MRSA-IV | N |
| 36 | M | 4 | wound swab | R | S | S | S | R | R | S | R | S | R | 24 | CIP-E-FD-SXT-TET | 6 | IV | t223 | *spa*-CC223/309 | CC22-MRSA-IV | N |
| 37 | F | 7 | wound swab | R | R | S | S | R | R | S | R | S | R | >256 | CIP-CN-E-FD-SXT-TET | 7 | IV | t064 | *spa*-CC008 | CC8-MRSA-IV | N |
| 38 | M | 18 | wound swab | S | S | S | S | R | R | S | S | S | R | 16 | E-FD-TET | 4 | IV | t127 | *spa*-CC127 | CC1-MRSA-IV | N |
| 39 | M | 0,17 | wound swab | R | S | R | S | R | R | S | S | S | R | 32 | CIP-DA-E-FD-TET | 6 | IV | t127 | *spa*-CC127 | CC1-MRSA-IV | N |
| 40 | F | 52 | wound swab | S | R | R | S | R | R | S | R | S | R | >256 | CN-DA-E-FD-SXT-TET | 7 | IV | t064 | *spa*-CC008 | CC8-MRSA-IV | N |
| 41 | M | 51 | wound swab | R | R | S | S | R | R | S | S | S | R | 4 | CIP-CN-E-FD-TET | 6 | IV | t008 | *spa*-CC008 | CC8-MRSA-IV | P |
| 42 | M | 2 | wound swab | R | R | R | R | R | R | S | R | S | R | >256 | CIP-CN-DA-DO-E-FD-SXT-TET | 8 | IV | t064 | *spa*-CC008 | CC8-MRSA-IV | N |
| 43 | F | 55 | wound swab | S | S | S | S | S | R | S | R | S | S | >256 | FD-SXT | 3 | IV | t064 | *spa*-CC008 | CC8-MRSA-IV | N |
| 44 | M | 18 | wound swab | R | R | S | S | S | R | S | R | S | S | >256 | CIP-CN-FD-SXT | 5 | IV | t064 | *spa*-CC008 | CC8-MRSA-IV | N |
| 45 | F | 5 | wound swab | R | R | S | S | S | S | S | R | S | S | 24 | CIP-CN-SXT | 4 | V | t657 | *spa*-CC657 | CC1/ST772-MRSA-V | P |
| 46 | F | 18 | wound swab | R | S | S | S | R | R | S | S | S | S | NA | CIP-E-FD | 4 | IV | t127 | *spa*-CC127 | CC1-MRSA-IV | N |
| 47 | F | 0,67 | wound swab | R | R | R | R | R | R | S | R | R | R | >256 | CIP-CN-DA-DO-E-FD-SXT-QD-TET | 9 | IV | t064 | *spa*-CC008 | CC8-MRSA-IV | N |
| 48 | M | 18 | wound swab | S | R | S | S | R | R | S | S | S | S | 16 | CN-E-FD | 4 | V | t657 | *spa*-CC657 | CC1/ST772-MRSA-V | P |
| 49 | M | 28 | wound swab | R | R | R | R | R | R | S | R | S | R | >256 | CIP-CN-DA-DO-E-FD-SXT-TET | 8 | IV | t064 | *spa*-CC008 | CC8-MRSA-IV | N |
| 50 | M | 25 | PUS | R | R | R | S | R | R | S | R | R | R | 32 | CIP-CN-DA-E-FD-SXT-QD-TET | 9 | II | t008 | *spa*-CC008 | CC8-MRSA-II | P |
| 51 | F | 45 | wound swab | R | S | S | S | S | S | S | S | S | S | >256 | CIP | 2 | V | t657 | *spa*-CC657 | CC1/ST772-MRSA-V | P |
| 52 | F | 25 | wound swab | S | S | S | S | R | R | S | S | R | R | 16 | E-FD-QD-TET | 5 | IV | t127 | *spa*-CC127 | CC1-MRSA-IV | N |
| 53 | M | 45 | wound swab | R | R | S | S | R | S | S | R | S | R | 8 | CIP-CN-E-SXT-TET | 6 | V | t345 | *spa*-CC657 | CC1/ST772-MRSA-V | P |
| 54 | F | 0,42 | wound swab | R | R | S | S | R | R | S | S | S | R | 32 | CIP-CN-E-FD-TET | 6 | IV | t525 | singleton | undetermined | N |
| 55 | M | 18 | wound swab | R | R | S | S | R | S | S | R | S | S | 16 | CIP-CN-E-SXT | 5 | V | t5414 | *spa*-CC657 | CC1/ST772-MRSA-V | P |
| 56 | M | 86 | wound swab | R | R | R | S | R | R | S | S | S | R | 32 | CIP-CN-DA-E-FD-TET | 7 | II | t008 | *spa*-CC008 | CC8-MRSA-II | P |
| 57 | F | 47 | wound swab | R | S | S | S | R | S | S | R | S | R | 32 | CIP-E-SXT-TET | 5 | IV | t127 | *spa*-CC127 | CC1-MRSA-IV | N |
| 58 | F | 13 | wound swab | R | R | S | S | R | S | S | R | S | S | 32 | CIP-CN-E-SXT | 5 | V | t657 | *spa*-CC657 | CC1/ST772-MRSA-V | P |
| 59 | F | 0,42 | wound swab | R | R | S | S | R | R | S | R | S | R | >256 | CIP-CN-E-FD-SXT-TET | 7 | IV | t064 | *spa*-CC008 | CC8-MRSA-IV | N |
| 60 | M | 15 | wound swab | R | R | R | S | R | S | S | R | S | S | 12 | CIP-CN-DA-E-SXT | 6 | V | t657 | *spa*-CC657 | CC1/ST772-MRSA-V | P |
| 61 | F | 29 | wound swab | R | R | R | S | R | R | S | R | S | S | 12 | CIP-CN-DA-E-FD-SXT | 7 | IV | t127 | *spa*-CC127 | CC1-MRSA-IV | N |
| 62 | F | 12 | wound swab | R | R | R | S | R | R | S | R | S | R | >256 | CIP-CN-DA-E-FD-SXT-TET | 8 | IV | t064 | *spa*-CC008 | CC8-MRSA-IV | N |
| 63 | M | 24 | wound swab | R | R | R | R | R | S | S | S | R | S | 24 | CIP-CN-DA-DO-E-QD | 7 | V | t5414 | *spa*-CC657 | CC1/ST772-MRSA-V | P |
| 64 | M | 18 | wound swab | R | S | S | S | R | S | S | R | S | S | 12 | CIP-E-SXT | 4 | III | t363 | singleton | undetermined | P |
| 65 | M | 18 | wound swab | R | R | R | S | R | R | S | R | S | R | 32 | CIP-CN-DA-E-FD-SXT-TET | 8 | IV | t127 | *spa*-CC127 | CC1-MRSA-IV | N |
| 66 | M | 30 | wound swab | R | R | R | S | R | R | S | R | S | R | 32 | CIP-CN-DA-E-FD-SXT-TET | 8 | IV | t127 | *spa*-CC127 | CC1-MRSA-IV | N |
| 67 | M | 10 | wound swab | R | R | R | S | R | R | S | R | S | R | >256 | CIP-CN-DA-E-FD-SXT-TET | 8 | IV | t064 | *spa*-CC008 | CC8-MRSA-IV | N |
| 68 | M | 61 | wound swab | R | R | S | S | R | R | S | R | S | R | 8 | CIP-CN-E-FD-SXT-TET | 7 | V | t657 | *spa*-CC657 | CC1/ST772-MRSA-V | P |
| 69 | M | 68 | wound swab | S | S | S | S | S | S | S | S | S | S | 24 | Susceptible | 1 | IV | t1309 | singleton | CC361-MRSA-IV | N |
| 70 | M | 51 | wound swab | R | R | S | S | S | S | S | R | S | S | 24 | CIP-CN-SXT | 4 | IV | t1309 | singleton | CC361-MRSA-IV | N |
| 71 | M | 16 | wound swab | R | R | R | S | R | R | S | R | S | R | 32 | CIP-CN-DA-E-FD-SXT-TET | 8 | IV | t064 | *spa*-CC008 | CC8-MRSA-IV | N |
| 72 | M | 37 | wound swab | R | R | S | S | R | R | S | R | S | S | 32 | CIP-DA-E-FD-SXT | 6 | V | t5414 | *spa*-CC657 | CC1/ST772-MRSA-V | P |
| 73 | F | 12 | wound swab | R | R | R | S | R | S | S | R | S | S | 8 | CIP-CN-DA-E-SXT | 6 | V | t657 | *spa*-CC657 | CC1/ST772-MRSA-V | P |
| 74 | M | 0,17 | wound swab | S | R | S | S | R | S | S | R | S | R | 8 | CN-E-SXT-TET | 5 | IV | t1749 | *spa*-CC021 | CC30-MRSA-IV | P |
| 75 | F | 16 | wound swab | R | R | S | R | R | R | S | R | S | S | 32 | CIP-CN-DO-E-FD-SXT | 7 | IV | t6100 | singleton | CC5-MRSA-IV | N |
| 76 | M | 14 | wound swab | R | R | R | S | R | S | S | R | S | S | 4 | CIP-CN-DA-E-SXT | 6 | IV | t6100 | singleton | CC5-MRSA-IV | N |
| 77 | F | 28 | wound swab | R | R | R | S | R | R | S | R | S | R | 8 | CIP-CN-DA-E-FD-SXT-TET | 8 | V | t021 | *spa*-CC021 | CC30-MRSA-V | P |
| 78 | M | 17 | wound swab | R | S | S | S | S | S | S | R | S | R | 32 | CIP-SXT-TET | 4 | IV | t127 | *spa*-CC127 | CC1-MRSA-IV | N |
| 79 | F | 41 | wound swab | R | R | S | S | R | S | S | S | S | S | 8 | CIP-CN-E | 4 | V | t657 | *spa*-CC657 | CC1/ST772-MRSA-V | P |
| 80 | F | 22 | wound swab | R | R | S | S | S | S | S | S | S | S | 32 | CIP-CN | 3 | V | t5414 | *spa*-CC657 | CC1/ST772-MRSA-V | P |
| 81 | M | 10 | wound swab | R | R | R | S | R | R | S | R | S | R | 48 | CIP-CN-DA-E-FD-SXT-TET | 8 | V | t5414 | *spa*-CC657 | CC1/ST772-MRSA-V | P |
| 82 | F | 22 | wound swab | R | R | R | S | R | R | S | R | S | S | 8 | CIP-CN-DA-E-FD-SXT | 7 | V | t657 | *spa*-CC657 | CC1/ST772-MRSA-V | P |
| 83 | F | 48 | wound swab | R | S | S | S | S | S | S | S | S | S | 4 | CIP | 2 | V | t127 | *spa*-CC127 | CC1-MRSA-V | N |
| 84 | M | 22 | wound swab | R | R | S | S | R | S | S | S | S | S | 8 | CIP-CN-E | 4 | V | t127 | *spa*-CC127 | CC1-MRSA-V | N |
| 85 | M | 72 | PUS | R | S | S | S | R | R | S | R | S | S | 8 | CIP-E-FD-SXT | 5 | V | t657 | *spa*-CC657 | CC1/ST772-MRSA-V | P |
| 86 | F | 24 | PUS | R | R | S | R | R | R | S | R | S | R | 12 | CIP-CN-DO-E-FD-SXT-TET | 7 | V | t657 | *spa*-CC657 | CC1/ST772-MRSA-V | P |
| 87 | M | 18 | wound swab | R | R | R | S | S | R | S | R | R | R | 32 | CIP-CN-DA-FD-SXT-QD-TET | 8 | IV | t127 | *spa*-CC127 | CC1-MRSA-IV | N |
| 88 | M | 10 | PUS | R | R | R | R | R | R | S | S | S | R | 24 | CIP-CN-DA-DO-E-FD-TET | 7 | III | t030 | *spa*-CC030/632 | CC8/ST239-MRSA-III | N |
| 89 | F | 63 | wound swab | R | R | S | S | R | R | S | R | S | S | 24 | CIP-DA-E-FD-SXT | 6 | IV | t127 | *spa*-CC127 | CC1-MRSA-IV | N |
| 90 | F | 18 | wound swab | R | R | R | S | R | R | S | S | S | R | 32 | CIP-CN-DA-E-FD-TET | 7 | IV | t127 | *spa*-CC127 | CC1-MRSA-IV | N |
| 91 | M | 18 | wound swab | R | S | S | S | S | S | S | S | S | S | 24 | CIP | 2 | IV | t6100 | singleton | CC5-MRSA-IV | N |
| 92 | M | 68 | wound swab | R | R | R | S | R | S | S | R | S | S | 8 | CIP-CN-DA-E-SXT | 6 | V | t657 | *spa*-CC657 | CC1/ST772-MRSA-V | P |
| 93 | F | 40 | wound swab | R | R | R | S | R | R | S | S | S | R | 32 | CIP-CN-DA-E-FD-TET | 7 | V | t5414 | *spa*-CC657 | CC1/ST772-MRSA-V | P |
| 94 | M | 35 | PUS | R | R | R | S | R | S | S | R | S | R | >256 | CIP-CN-DA-E-SXT-TET | 7 | III | t632 | *spa*-CC030/632 | CC8/ST239-MRSA-III | N |
| 95 | F | 18 | wound swab | R | R | R | S | R | S | S | R | S | S | 6 | CIP-CN-DA-E-SXT | 6 | V | t657 | *spa*-CC657 | CC1/ST772-MRSA-V | P |
| 96 | M | 18 | wound swab | R | R | R | R | R | R | S | R | S | R | >256 | CIP-CN-DA-DO-E-FD-SXT-TET | 8 | IV | t064 | *spa*-CC008 | CC8-MRSA-IV | N |
| 97 | F | 40 | wound swab | R | R | R | S | R | S | S | R | S | R | 16 | CIP-CN-DA-E-SXT-TET | 7 | IV | t304 | *spa*-CC008 | CC8-MRSA-IV | N |
| 98 | M | 63 | wound swab | R | R | R | S | R | R | S | R | S | R | 12 | CIP-CN-DA-E-FD-SXT-TET | 8 | IV | t021 | *spa*-CC021 | CC30-MRSA-IV | P |
| 99 | F | 61 | wound swab | R | R | R | S | R | R | S | S | S | R | 12 | CIP-CN-DA-E-FD-TET | 7 | IV | t127 | *spa*-CC127 | CC1-MRSA-IV | N |
| 100 | M | 55 | wound swab | R | S | S | R | R | S | S | R | S | S | 16 | CIP-DO-E-SXT | 5 | V | t657 | *spa*-CC657 | CC1/ST772-MRSA-V | P |
| 101 | F | 49 | wound swab | R | R | R | S | R | R | S | S | S | S | 8 | CIP-CN-DA-E-FD | 6 | V | t657 | *spa*-CC657 | CC1/ST772-MRSA-V | P |
| 102 | F | 45 | wound swab | R | R | R | S | R | R | S | S | S | R | 16 | CIP-CN-DA-E-FD-TET | 7 | IV | t1784 | excluded | CC1-MRSA-IV | N |
| 103 | M | 12 | wound swab | R | R | R | R | R | R | S | R | S | R | >256 | CIP-CN-DA-DO-E-FD-SXT-TET | 8 | IV | t064 | *spa*-CC008 | CC8-MRSA-IV | N |
| 104 | F | 45 | wound swab | R | S | R | S | R | R | S | S | S | R | 12 | CIP-DA-E-FD-TET | 6 | IV | t177 | *spa*-CC127 | CC1-MRSA-IV | N |
| 105 | F | 76 | wound swab | R | S | R | R | R | R | S | S | S | R | 16 | CIP-DA-DO-E-FD-TET | 6 | IV | t127 | *spa*-CC127 | CC1-MRSA-IV | N |
| 106 | M | 18 | PUS | R | R | S | R | R | R | S | R | S | R | 6 | CIP-CN-DO-E-FD-SXT-TET | 7 | IV | t657 | *spa*-CC657 | CC1/ST772-MRSA-IV | P |
| 107 | M | 3 | wound swab | R | S | S | R | R | S | S | S | S | R | 24 | CIP-DO-E-TET | 4 | V | t127 | *spa*-CC127 | CC1-MRSA-V | N |
| 108 | F | 28 | wound swab | R | R | R | R | R | R | S | S | S | R | 24 | CIP-CN-DA-DO-E-FD-TET | 7 | V | t127 | *spa*-CC127 | CC1-MRSA-V | N |
| 109 | M | 5 | PUS | R | R | R | S | R | S | S | R | S | S | 8 | CIP-CN-DA-E-SXT | 6 | IV | t345 | *spa*-CC657 | CC1/ST772-MRSA-IV | P |
| 110 | M | 18 | wound swab | R | R | R | R | R | R | S | R | S | R | 256 | CIP-CN-DA-DO-E-FD-SXT-TET | 8 | IV | t064 | *spa*-CC008 | CC8-MRSA-IV | N |
| 111 | M | 55 | wound swab | R | R | R | S | R | R | S | R | S | R | 4 | CIP-CN-DA-E-FD-SXT-TET | 8 | V | t127 | *spa*-CC127 | CC1-MRSA-V | N |
| 112 | M | 55 | wound swab | R | R | S | S | R | S | S | R | S | S | 24 | CIP-CN-E-SXT | 5 | IV | t309 | *spa*-CC223/309 | CC22-MRSA-IV | N |
| 113 | F | 30 | wound swab | R | S | R | S | R | R | S | R | S | S | 16 | CIP-DA-E-FD-SXT | 6 | IV | t309 | *spa*-CC223/309 | CC22-MRSA-IV | N |
| 114 | F | 40 | wound swab | R | R | R | S | R | R | S | R | S | R | 16 | CIP-CN-DA-E-FD-SXT-TET | 8 | IV | t127 | *spa*-CC127 | CC1-MRSA-IV | N |
| 115 | M | 17 | PUS | R | R | R | S | R | S | S | R | S | S | 32 | CIP-CN-DA-E-SXT | 6 | IV | t363 | singleton | undetermined | P |
| 116 | F | 24 | PUS | R | R | S | R | R | R | S | R | S | R | 24 | CIP-CN-DO-E-FD-SXT-TET | 7 | IV | t657 | *spa*-CC657 | CC1/ST772-MRSA-IV | P |
| 117 | M | 4 | wound swab | R | S | S | S | R | R | S | S | S | R | 24 | CIP-E-FD-TET | 5 | IV | t127 | *spa*-CC127 | CC1-MRSA-IV | N |
| 118 | M | 50 | wound swab | R | R | S | S | R | S | S | S | S | S | >256 | CIP-CN-E | 4 | V | t657 | *spa*-CC657 | CC1/ST772-MRSA-V | P |
| 119 | M | 13 | wound swab | R | R | R | R | R | R | S | S | S | R | 8 | CIP-CN-DA-DO-E-FD-TET | 7 | IV | t657 | *spa*-CC657 | CC1/ST772-MRSA-IV | P |
| 120 | F | 20 | wound swab | R | S | S | R | R | S | S | R | S | R | 8 | CIP-DO-E-SXT-TET | 5 | IV | t304 | *spa*-CC008 | CC8-MRSA-IV | N |
| 121 | M | 71 | wound swab | R | R | S | R | S | S | S | R | S | R | 24 | CIP-CN-DO-SXT-TET | 5 | IV | t064 | *spa*-CC008 | CC8-MRSA-IV | N |
| 122 | M | 9 | PUS | R | S | S | R | S | S | S | R | S | R | 32 | CIP-DO-SXT-TET | 4 | IV | t345 | *spa*-CC657 | CC1/ST772-MRSA-IV | P |
| 123 | F | 66 | PUS | R | R | S | S | R | S | S | R | S | S | 24 | CIP-CN-E-SXT | 5 | V | t5414 | *spa*-CC657 | CC1/ST772-MRSA-V | P |
| 124 | F | 49 | PUS | S | S | S | S | R | R | S | R | S | S | 16 | E-FD-SXT | 4 | V | t304 | *spa*-CC008 | CC8-MRSA-V | N |
| 125 | M | 2 | PUS | R | S | R | S | R | S | S | R | R | R | 32 | CIP-DA-E-SXT-QD-TET | 7 | V | t304 | *spa*-CC008 | CC8-MRSA-V | N |
| 126 | F | 0,42 | wound swab | R | S | R | S | R | S | S | R | S | R | 32 | CIP-DA-E-SXT-TET | 6 | IV | t657 | *spa*-CC657 | CC1/ST772-MRSA-IV | P |
| 127 | M | 32 | wound swab | R | R | R | R | R | R | S | R | S | R | 24 | CIP-CN-DA-DO-E-FD-SXT-TET | 8 | IV | t064 | *spa*-CC008 | CC8-MRSA-IV | N |
| 128 | F | 50 | PUS | R | R | S | R | R | R | S | S | S | S | 12 | CIP-CN-DO-E-FD | 6 | V | t127 | *spa*-CC127 | CC1-MRSA-V | N |
| 129 | M | 10 | wound swab | R | S | S | R | R | R | S | S | R | R | 16 | CIP-DO-E-FD-QD-TET | 6 | IV | t114 | *spa*-CC127 | CC1-MRSA-IV | N |
| 130 | F | 5 | wound swab | R | R | S | R | R | R | S | R | S | R | >256 | CIP-CN-DO-E-FD-SXT-TET | 7 | IV | t064 | *spa*-CC008 | CC8-MRSA-IV | N |
| 131 | M | 40 | wound swab | R | R | R | S | R | S | S | R | S | R | 32 | CIP-CN-DA-E-SXT-TET | 7 | IV | t064 | *spa*-CC008 | CC8-MRSA-IV | N |
| 132 | F | 6 | wound swab | R | R | S | S | R | R | S | R | S | S | 32 | CIP-DA-E-FD-SXT | 6 | V | t657 | *spa*-CC657 | CC1/ST772-MRSA-V | P |
| 133 | F | 4 | wound swab | R | R | S | R | R | R | S | R | S | R | 8 | CIP-CN-DO-E-FD-SXT-TET | 7 | IV | t064 | *spa*-CC008 | CC8-MRSA-IV | N |
| 134 | F | 2 | wound swab | R | R | R | S | R | R | S | R | S | R | 16 | CIP-CN-DA-E-FD-SXT-TET | 8 | IV | t064 | *spa*-CC008 | CC8-MRSA-IV | N |
| 135 | F | 65 | wound swab | S | S | S | S | S | R | S | R | S | S | 32 | FD-SXT | 3 | V | t030 | *spa*-CC030/632 | CC8/ST239-MRSA-V | N |
| 136 | M | 18 | wound swab | R | R | S | S | R | R | S | S | S | S | 16 | CIP-CN-E-FD | 5 | IV | t008 | *spa*-CC008 | CC8-MRSA-IV | P |
| 137 | M | 3 | PUS | S | R | S | S | R | S | S | R | S | S | 32 | CN-E-SXT | 4 | V | t3596 | *spa*-CC657 | CC1/ST772-MRSA-V | P |
| 138 | M | 45 | wound swab | R | R | S | S | R | S | S | R | S | S | 24 | CIP-CN-E-SXT | 5 | V | t5414 | *spa*-CC657 | CC1/ST772-MRSA-V | P |
| 139 | F | 25 | wound swab | R | R | R | R | R | R | S | R | S | R | 24 | CIP-CN-DA-DO-E-FD-SXT-TET | 8 | IV | t064 | *spa*-CC008 | CC8-MRSA-IV | N |
| 140 | M | 10 | wound swab | R | S | S | R | R | S | S | S | S | R | 8 | CIP-DO-E-TET | 4 | IV | t127 | *spa*-CC127 | CC1-MRSA-IV | N |
| 141 | M | 23 | wound swab | S | S | S | S | S | S | S | R | S | S | 8 | SXT | 2 | III | t987 | *spa*-CC021 | CC8/ST239-MRSA-III | N |
| 142 | M | 10 | wound swab | R | R | R | S | R | R | S | R | S | R | 32 | CIP-CN-DA-E-FD-SXT-TET | 8 | V | t657 | *spa*-CC657 | CC1/ST772-MRSA-V | P |
| 143 | M | 37 | wound swab | R | R | S | S | R | R | S | R | S | S | 24 | CIP-DA-E-FD-SXT | 6 | V | t021 | *spa*-CC021 | CC30-MRSA-V | P |
| 144 | M | 18 | wound swab | R | R | R | R | R | R | S | R | S | R | 16 | CIP-CN-DA-DO-E-FD-SXT-TET | 8 | V | t657 | *spa*-CC657 | CC1/ST772-MRSA-V | P |
| 145 | F | 49 | PUS | S | S | S | S | R | R | S | R | S | S | 32 | E-FD-SXT | 4 | V | t021 | *spa*-CC021 | CC30-MRSA-V | P |
| 146 | M | 60 | PUS | R | R | S | S | S | S | S | R | S | R | 32 | CIP-CN-SXT-TET | 5 | IV | t127 | *spa*-CC127 | CC1-MRSA-IV | N |
| 147 | M | 5 | PUS | R | R | R | S | S | S | S | R | S | S | 32 | CIP-CN-DA-SXT | 5 | IV | t064 | *spa*-CC008 | CC8-MRSA-IV | N |
| 148 | M | 18 | wound swab | R | S | S | S | S | S | S | S | S | S | 24 | CIP | 2 | IV | t127 | *spa*-CC127 | CC1-MRSA-IV | N |
| 149 | F | 45 | wound swab | R | S | S | S | S | S | S | S | S | S | 16 | CIP | 2 | V | t657 | *spa*-CC657 | CC1/ST772-MRSA-V | P |
| 150 | M | 50 | wound swab | R | R | S | S | R | S | S | S | S | S | 16 | CIP-CN-E | 4 | V | t273 | singleton | CC1-MRSA-V | P |
| 151 | F | 41 | wound swab | R | R | S | S | S | S | S | S | S | S | 8 | CIP-CN | 3 | IV | t008 | *spa*-CC008 | CC8-MRSA-IV | P |
| 152 | F | 22 | wound swab | R | R | S | S | S | S | S | S | S | S | 8 | CIP-CN | 3 | V | t4109 | *spa*-CC021 | CC30-MRSA-V | P |
| 153 | M | 71 | wound swab | R | R | S | S | S | R | S | R | S | R | 24 | CIP-CN-FD-SXT-TET | 6 | V | t5414 | *spa*-CC657 | CC1/ST772-MRSA-V | P |
| 154 | M | 42 | wound swab | S | R | R | R | R | R | S | R | S | R | 8 | CN-DA-DO-E-FD-SXT-TET | 7 | IV | t064 | *spa*-CC008 | CC8-MRSA-IV | N |

The supplementary table summarises characteristics of all isolates.

Abbreviation:

**Gender**: M, male; F, female.

**Antimicrobials**: R, resistant; S, susceptible; CIP, ciprofloxacin; CN, gentamicin; DA, clindamycin; DO, doxycycline; E, erythromycin; FD, fusidic acid; SXT, sulphamethoxazole; QD, quinupristin/dalfopristin; TET, tetracycline; MIC, minimum inhibitory concentration. No isolate was resistant to linezolid.

* The quantification of the multiresistance was based on the categorisation of antimicrobial classes used for the definition of multidrug-resistance in *S. aureus* according Magiorakos et al. [11]. Multiresistance is defined as non-susceptibility to at least one agent in three or more antimicrobial categories. The numbers represent the number of antimicrobial categories to which the isolates were resistant.

***spa*-CC**, spa clonal complex.

**PVL** (Panton-Valentine leucocidin): P, positive; N, negative.
